# Care challenges in older general hospital patients: Impact of cognitive impairment and other patient-related factors

**DOI:** 10.1007/s00391-019-01628-x

**Published:** 2019-10-18

**Authors:** Ingrid Hendlmeier, Horst Bickel, Johannes Baltasar Heßler-Kaufmann, Martina Schäufele

**Affiliations:** 1 Faculty of Social Sciences, Hochschule Mannheim, University of Applied Sciences, Mannheim, Germany; 2 Working Group Psychiatric Epidemiology, Department of Psychiatry and Psychotherapy, Technical University of Munich (TUM), Klinikum rechts der Isar, Munich, Germany

**Keywords:** Cognitive impairment, Cross-sectional studies, Adverse events, Nursing, Dementia, Kognitive Beeinträchtigung, Querschnittsstudie, Unerwünschte Ereignisse, Pflege, Demenz

## Abstract

**Background:**

Older general hospital patients, particularly those with cognitive impairment, frequently experience adverse events and other care complications during their stay. As these findings have so far been based on small and selected patient samples, the aim of the present study was to provide reliable data on a) the prevalence of adverse care issues (summarized under the term care challenges) in older general hospital patients and on b) associated patient-related risk factors (e.g. cognitive impairment).

**Methods:**

A cross-sectional representative study comprising 1469 patients aged ≥65 years from 33 randomly selected general hospitals in southern Germany (GHoSt). Data collection included the use of different data sources, e.g. structured interviews with responsible nursing staff concerning care challenges and procedures for determining the patients’ cognitive status.

**Results:**

Care challenges were statistically significantly (*p* < 0.001) more often reported for patients with dementia and/or delirium (87.5%) and mild cognitive impairment (47.9%) compared to cognitively unimpaired patients (24.6%). Adjusted odds ratios suggested cognitive impairment, impaired activities of daily living, receiving long-term care and unplanned admission as significant patient-related risk factors for care challenges. Furthermore, the occurrence of such issues was associated with the application of physical restraints, support from relatives, prescription of psycholeptics and specialist consultations.

**Conclusion:**

The findings suggest a strong impact of different degrees of cognitive impairment on challenges in care. The results might help to design appropriate training programs for hospital staff and other interventions to prevent or reduce critical situations.

## Zusammenfassung

**Hintergrund:**

Für ältere Allgemeinkrankenhauspatienten werden häufig unerwünschte Ereignisse und Probleme in der Versorgung während ihres Aufenthaltes berichtet, v. a. bei kognitiv beeinträchtigten Patienten. Die Befunde basieren jedoch auf kleinen und selektiven Stichproben. Ziel dieser Studie ist es, zuverlässige Daten a) zur Prävalenz solcher Versorgungsprobleme (zusammengefasst als „herausfordernde Pflegesituationen“) und b) zu assoziierten patientenbezogenen Risikofaktoren (v. a. kognitive Beeinträchtigung) zu gewinnen.

**Methoden:**

Die repräsentative Querschnittsstudie umfasst 1469 65-jährige und ältere Patienten in 33 zufällig ausgewählten Allgemeinkrankenhäusern in Süddeutschland (General Hospital Study, GHoSt). Informationsquellen waren u. a. eine standardisierte Befragung der jeweils verantwortlichen Pflegefachkraft und Verfahren zur Feststellung des kognitiven Status.

**Ergebnisse:**

Im Vergleich zu kognitiv unbeeinträchtigten Patienten (24,6 %) wurden herausfordernde Pflegesituationen statistisch signifikant (*p* < 0,001) häufiger bei Patienten mit Demenz/Delir (87,5 %) und mit leichter kognitiver Beeinträchtigung (47,9 %) berichtet. Adjustierte „odds ratios“ bestätigten kognitive Beeinträchtigung, eingeschränkte Alltagskompetenz, Bezug von Pflegeleistungen und ungeplante Aufnahme als Risikofaktoren. Versorgungsprobleme waren zudem häufiger assoziiert mit freiheitsentziehenden Maßnahmen, Angehörigeneinbindung in die Pflege, Verordnung von Psycholeptika und Konsilien.

**Diskussion:**

Die Befunde belegen den deutlichen Einfluss verschiedener Grade kognitiver Beeinträchtigung auf herausfordernde Pflegesituationen. Die Ergebnisse können dazu beitragen, geeignete Schulungsprogramme und Interventionen zu konzipieren, um kritische Situationen zu vermeiden oder zu minimieren.

## Background

Adverse events (e.g. falls) and other care complications (e.g. noncompliant behavior) may render hospital stays of older adults distressing and lead to serious consequences, not only for the patients themselves but also for their relatives and hospital staff [9]. While these occurrences are associated with secondary harm to patient health, increased length of stay and other negative consequences [3, 4, 18, 32], they also pose substantial challenges and stress to hospital staff [12, 21]. In particular, wandering, activity disturbances, aggression, sleep disturbances, and fending off help with eating showed a significant impact on staff daily routines [28, 29]. The relevance of these problems seems to increase in the presence of cognitive impairment [13, 28, 32].

Reliable key data about the frequency and distribution of adverse care issues are essential, given the pressing need to improve the quality of hospital care of older patients. As the previous findings were based on international studies with small and highly selected samples, it is unclear how common these care problems really are and whether the available findings also pertain to general hospitals in Germany. In addition, the applied methods and definitions of adverse events, care problems, and care complications varied widely across studies.

The aim of this study was to determine a) the prevalence of care challenges in older general hospital patients and b) the associations of these challenges with different degrees of cognitive impairment and other patient-related risk factors (e.g. demographics, degree of functional impairment). The term care challenges summarizes a variety of adverse events and other care issues on the level of everyday experiences of patients and hospital staff.

## Material and methods

### Sampling

Data were taken from the General Hospital Study (GHoSt), which is a cross-sectional representative study of patients aged ≥65 years in randomly selected general hospitals in southern Germany [2, 19]. There were inclusion and exclusion criteria on the hospital level, ward level and patient level. Small hospitals (<150 beds), specialized hospitals (e.g. psychiatric clinics), rehabilitation and day or night clinics were excluded. On the ward level, intensive care units, isolation, pediatric, geriatric, neurological and psychiatric wards were not considered. Exclusion criteria on the patient level were age under 65 years, critical condition, isolation because of an infectious disease, and insufficient proficiency in the German language.

A multistep sampling procedure was applied. First, all general hospitals meeting the inclusion criteria were put into a random order, contacted according to this order and asked for participation until the previously set number of 33 hospitals were achieved. Second, in each participating hospital five wards and one substitute ward meeting the criteria were randomly chosen. Trained research assistants visited one ward each day in the survey week. They asked all inpatients fulfilling the inclusion criteria or their legal representatives for informed consent to participate in the study.

The ethics committee of the Faculty of Medicine of the Technical University of Munich approved the study protocol (No. 66/14) and the study was registered in the German Registry of Clinical Studies under DRKS00006028. The survey was conducted between June 2014 and May 2015.

### Data collection

On the survey day the research assistants a) asked each patient or a respective knowledgeable informant about demographic data, b) conducted a structured bedside examination (for more details see [2]), c) conducted a standardized interview for each participating patient with the responsible nurse concerning the patient’s status of activities of daily living (ADL), psychosocial characteristics (e.g. visits and support from relatives), medical and care features (e.g. anesthesia, application of physical restraints), and care challenges observed since patient admission and d) collected relevant information from each patient’s medical records, e.g. admission reason, prescription of psycholeptics (group N05 according to Anatomic Therapeutic Chemical classification system [ATC]) and analgesics (ATC group N02). The selection of care challenges (see Table 2) was based on previous reports of general hospital staff [12, 24, 29, 31]. As the items were based on the everyday experience of the hospital staff rather than on clinical concepts, the ratings can be assumed to have a higher reliability [26].

Global severity of cognitive impairment was rated with the clinical dementia rating scale (CDR [22]). Patients with a CDR score of 0 were considered to be cognitively unimpaired and patients with a CDR score of 0.5 to have mild cognitive impairment. Patients with CDR score of 1 (mild dementia and/or delirium), 2 (moderate dementia and/or delirium) or 3 (severe dementia and/or delirium) were summarized under the term dementia and/or delirium. Dementia was diagnosed according to Diagnostic and Statistical Manual of Mental Disorders IV (DSM-IV) criteria and delirium was assessed by means of the confusion assessment method [23].

### Statistical analysis

The care challenge items were dichotomized to present vs. not present. The 95% confidence intervals (CI) of the frequencies were calculated according to the Wilson score method [7]. Differences between the examined patient groups were assessed with analyses of variance (ANOVA), Pearson’s χ^2^-tests or Fisher’s exact tests. Associations between ≥1 reported care challenge and patient-related risk factors, e.g. age, gender, cognitive status, residential situation, receiving long-term care (LTC) benefits, unplanned admission, department and ADL were examined with univariate binary logistic regressions analyses. Multivariate logistic regression analysis was calculated with patient-related risk factors as predictors entered in one block. The statistically significantly predictors from this analysis were then used as covariates in multivariate logistic regression analyses examining the association of severity of cognitive impairment with the occurrence of specific care challenges as individual outcomes. In addition, univariate binary logistic regressions analyses were calculated with care and medical treatment characteristics to examine the associations with ≥1 reported care challenge. Using logistic generalized estimation equation (GEE) models ensured taking the cluster structure of the study into account.

## Results

The participation rate of the hospitals was 60% (33 out of 55) and the response rate on the patient level was 68.2%. On the specific days of survey 2534 patients aged 65 years or older were registered on the visited wards: 380 patients could not be asked for participation as they were isolated, repeatedly not present, in a critical condition or out of other reasons and 685 patients refused participation or their legal representative refused or could not be reached. One patient was excluded because there was insufficient information to determine the cognitive status with the CDR. The final sample consisted of 1468 patients aged 65–105 years (mean 78.6 years, SD 7.4 years) and 53.8% were female. The median time of inpatient stay to survey day was 5 days (range 1–95 days, interquartile range 2–9 days). The three cognitive impairment groups differed with respect to age, receiving LTC benefits, need of assistance in basic care, residential situation, unplanned admission, prescription of psycholeptics, application of physical restraints, and support from relatives (Table 1).

**Table 1:** Characteristics of the patients aged ≥65 years according to the severity of cognitive impairment assessed by clinical dementia rating scale (CDR)

		All patients (*n* = 1468)			Severity of cognitive impairment in hospital									*Significance*		
					Dementia and/or delirium CDR^a^ = 1–3 (*n* = 297)			Mild cognitive impairment CDR^a^ = 0.5 (*n* = 290)			No cognitive impairment CDR^a^ = 0 (*n* = 881)			*F*^b^	χ^2c^	*p*-value
*Age (years)*	*n, mean, SD*	1468	78.6	*7.4*	297	**83.2**	*7.1*	290	**80.7**	*6.7*	881	**76.4**	*6.9*	*122.5*	–	*0.000*
*Sex, female*	*n, %, 95% CI*	790	53.8	*51.3–56.4*	163	54.9	*49.2–60.4*	161	55.5	*49.8–61.1*	466	52.9	*49.6–56.2*	–	*0.78*	*0.679*
*Residential situation …*																
Nursing home	*n, %, 95% CI*	119	8.1	*6.8–9.6*	83	**28.1**	*23.3–33.4*	17	**5.9**	*3.7–9.2*	19	**2.2**	*1.4–3.3*	–	*202.45*	*0.000*
Community dwelling	*n, %, 95% CI*	1347	91.9	*90.4–93.2*	212	**71.9**	*66.5–76.7*	273	**94.1**	*90.8–96.3*	862	**97.8**	*96.7–98.6*			
*Receiving long-term care benefits*	*n, %, 95% CI*	361	24.6	*22.5–26.9*	168	**56.9**	*51.3–62.5*	88	**30.4**	*25.4–36.0*	105	**11.9**	*9.9–14.2*	–	*247.9*	*0.000*
*Unplanned admission*	*n, %, 95% CI*	737	50.5	*48.0–53.1*	189	**64.1**	*58.4–69.3*	157	**54.5**	*48.7–60.2*	391	**44.6**	*41.4–47.9*	–	*35.64*	*0.000*
*Staying at …*																
Internal medicine department	*n, %, 95% CI*	736	50.1	*47.6–52.7*	175	**58.9**	*53.3–64.5*	158	**54.5**	*48.7–60.1*	403	**45.7**	*42.5–49.0*	–	*25.07*	*0.000*
Surgical department	*n, %, 95% CI*	535	36.4	*34.0–38.9*	98	**33.0**	*27.9–38.5*	103	**35.5**	*30.2–41.2*	334	**37.9**	*34.8–41.8*	–		
Other department	*n, %, 95% CI*	197	13.4	*11.8–15.3*	24	**8.1**	*5.5–11.7*	29	**10.0**	*7.1–14.0*	144	**16.3**	*14.1–18.9*	–		
*Assistance in basic activities of daily living (ADL)*^d^	*n, %, 95% CI*	902	62.3	*59.8–64.8*	276	**95.8**	*92.9–97.6*	199	**70.6**	*65.0–75.6*	427	**48.6**	*45.3–51.9*	–	*215.9*	*0.000*
*ADL*^e^* (0–85, lower score indicates more need of basic nursing)*	*n, mean, SD*	1448	64.2	*24.6*	288	**36.4**	*25.3*	282	**61.7**	*22.7*	878	**74.1**	*16.4*	*395.6*	–	*0.000*
*Received general anesthesia*	*n, %, 95% CI*	534	36.6	*34.2–39.1*	82	**27.7**	*22.9–33.1*	100	**34.7**	*29.5–40.4*	352	**40.2**	*37.0–43.5*	–	*15.5*	*0.000*
*Psycholeptics prescription (ATC*^f^* N05)*	*n, %, 95% CI*	327	22.5	*20.4–24.7*	116	**39.5**	*34.0–45.1*	75	**26.1**	*21.4–31.5*	136	**15.6**	*13.3–18.2*	–	*74.5*	*0.000*
*Analgesics prescription (ATC*^f^* N02)*	*n, %, 95% CI*	807	55.0	*52.4–57.5*	150	50.5	*44.9–56.2*	160	55.2	*49.4–60.8*	497	56.4	*53.1–59.7*	–	*3.14*	*0.208*
*Physical restraints*	*n, %, 95% CI*	90	6.1	*5.0–7.5*	74	**24.9**	*20.3–30.1*	13	**4.5**	*2.6–7.5*	3	**0.3**	*0.1–1.0*	–	*234.8*	*0.000*
*Visit(s) of relatives/others*^h^	*n, %, 95% CI*	957	69.9	*67.4–72.3*	199	69.8	*64.3–74.9*	188	72.0	*66.3–77.1*	570	69.3	*66.0–72.3*	–	*0.73*	*0.696*
*Getting help from relatives in hospital*	*n, %, 95% CI*	178	13.1	*11.4–15.0*	90	**32.0**	*26.9–37.7*	42	**16.2**	*12.2–21.2*	46	**5.6**	*4.2–7.4*	–	*131.4*	*0.000*
*Geriatic/neurological/psychiatric consultation*	*n, %, 95% CI*	92	6.3	*5.2–7.7*	38	**12.9**	*9.5–17.2*	15	**5.2**	*3.2–8.4*	39	**4.5**	*3.3–6.1*	–	*27.12*	*0.000*

^*a*^*CDR *clinical dementia rating scale

^b^ANOVA

^c^Pearson’s χ^2^-test

^d^Assistance means less than 85 points in modified Barthel index (items bathing and climbing stairs were excluded)

^e^Modified Barthel index 0–85 points (items bathing and climbing stairs were excluded)

^*f*^*ATC* anatomical therapeutic chemical classification

^g^Unwanted bedside rail and other physical restraints

^h^Frequent visits

Any care challenge was reported for 42% of the total sample (Table 2). The responsible nurses reported needing more time for caring than usual in 20% of the patients. In addition, 18% of the patients showed sleep disturbances and 17.4% were fending off medical nursing or forgot to take their medication. Limited communication ability was reported for 13.7% of the patients, implying that they were not able to understand questions, follow instructions and/or express their needs and wishes.

**Table 2:** Care challenges among patients aged ≥65 years according to the severity of cognitive impairment (CDR^a^)

	All patients (*n* = 1468)			Severity of cognitive impairment in hospital									Significance	
				Dementia and/or delirium CDR^a^ = 1–3 (*n* = 297)			Mild cognitive impairment CDR^a^ = 0.5 (*n* = 290)			No cognitive impairment CDR^a^ = 0 (*n* = 881)			*χ*^*2*^^b^	*p*
	*n*	*%*	*95% CI*	*n*	*%*	*95% CI*	*n*	*%*	*95% CI*	*n*	*%*	*95% CI*		
≥1 care challenge	616	42.0	*39.5–44.5*	260	87.5	*83.3–90.8*	139	47.9	*42.3–53.7*	217	24.6	*21.9–27.6*	*366.3*	*0.000*
Needing more time than usual	293	20.0	*18.1–22.1*	168	56.9	*51.3–62.5*	57	19.8	*15.6–24.8*	68	7.7	*6.1–9.7*	*334.2*	*0.000*
Sleeping disturbance	257	18.0	*16.1–20.1*	120	42.1	*36.5–47.9*	57	20.4	*16.1–25.5*	80	9.3	*7.5–11.4*	*157.1*	*0.000*
Fending off medical nursing	255	17.4	*15.6–19.4*	159	53.7	*48.0–59.3*	45	15.5	*11.8–20.1*	51	5.8	*4.4–7.6*	*354.5*	*0.000*
Fending off/forgetting medication	208	14.2	*12.5–16.1*	126	42.9	*37.3–48.6*	39	13.4	*10.0–17.9*	43	4.9	*3.7–6.5*	*260.5*	*0.000*
Fending off wound care	48	3.3	*2.5–4.3*	36	12.2	*9.0–16.4*	5	1.7	*0.7–4.0*	7	0.8	*0.4–1.6*	*93.5*	*0.000*
Pulling out infusion needles, catheters etc.	69	4.7	*3.7–5.9*	59	19.9	*15.8–24.9*	5	1.7	*0.7–4.0*	5	0.6	*0.2–1.3*	*192.4*	*0.000*
Limited communication ability	201	13.7	*12.1–15.6*	156	52.9	*47.2–58.5*	18	6.2	*4.0–9.6*	27	3.1	*2.1–4.4*	*480.0*	*0.000*
Fending off basic nursing	128	8.8	*7.4–10.3*	81	27.5	*22.7–32.8*	22	7.6	*5.1–11.3*	25	2.8	*1.9–4.2*	*168.1*	*0.000*
Problem with eating and drinking	101	6.9	*5.9–8.6*	66	22.4	*18.1–27.6*	20	7.0	*4.6–10.6*	15	1.7	*1.0–2.8*	*146.9*	*0.000*
Leaving food/drinks untouched	76	5.2	*4.2–6.5*	42	14.2	*10.7–18.7*	19	6.6	*4.3–10.1*	15	1.7	*1.04–2.8*	*71.7*	*0.000*
Fending off help for eating/drinking	42	2.9	*2.1–3.9*	40	13.6	*10.2–18.0*	2	0.7	*0.2–2.5*	0	0	–	*152.3*	*0.000*
Throwing food	11	0.8	*0.4–1.3*	11	3.7	*2.1–6.6*	0	0	–	0	0	–	*30.8*^c^	*0.000*^c^
Complaints from other patients	93	6.3	*5.2–7.7*	47	15.8	*12.1–20.4*	25	8.6	*5.9–12.4*	21	2.4	*1.6–3.6*	*70.6*	*0.000*
Ringing bell without recognizable purpose	95	6.5	*5.3–7.9*	45	15.2	*11.5–19.7*	23	8.0	*5.4–11.7*	27	3.1	*2.1–4.4*	*54.9*	*0.000*
Being verbally agitated	89	6.1	*5.0–7.4*	58	19.5	*15.4–24.4*	18	6.2	*4.0–9.6*	13	1.5	*0.9–2.5*	*127.1*	*0.000*
Shouting for help	65	4.4	*3.5–5.6*	52	17.5	*13.6–22.2*	9	3.1	*1.6–5.8*	4	0.5	*0.2–1.2*	*154.2*	*0.000*
Insulting others	48	3.3	*2.5–4.3*	29	9.8	*6.9–13.8*	9	3.1	*1.6–5.8*	10	1.1	*0.6–2.1*	*52.8*	*0.000*
Problem in physician treatment	76	5.2	*4.2–6.5*	42	14.4	*10.2–18.9*	10	3.5	*1.9–6.3*	24	2.7	*1.8–4.0*	*62.4*	*0.000*
Not following physician instruction	50	3.5	*2.6–4.5*	30	10.4	*7.3–14.3*	7	2.4	*1.2–4.9*	13	1.4	*0.9–2.5*	*52.5*	*0.000*
Fending off physician treatment	40	2.7	*2.0–3.7*	20	6.9	*4.5–10.4*	6	2.1	*1.0–4.4*	14	1.6	*1.0–2.8*	*23.5*	*0.000*
Getting injured by falling	15	1.0	*0.6–1.7*	8	2.7	*1.4–5.2*	3	1.0	*0.4–3.0*	4	0.5	*0.2–1.2*	*9.5*^c^	*0.006*^c^
Wandering	45	3.1	*2.3–4.1*	36	12.1	*8.9–16.3*	6	2.1	*01.0–4.5*	3	0.3	*0.1–1.0*	*104.7*	*0.000*
Being physically aggressive	40	2.7	*2.0–3.7*	36	12.1	*8.9–16.3*	3	1.0	*0.4–3.0*	1	0.1	*0.0–0.6*	*124.7*	*0.000*
Leaving ward/hospital unnoticed	23	1.6	*1.1–2.4*	20	6.8	*4.4–10.2*	3	1.0	*0.4–3.0*	0	0	–	*53.6*^c^	*0.000*^c^

^*a*^*CDR *clinical dementia rating scale

^b^Pearson’s χ^2^-test

^c^Fisher’s exact test

For half of the patients with mild cognitive impairment and for 87.5% of patients with dementia and/or delirium nursing staff reported at least 1 care challenge compared to 24.6% in the cognitively unimpaired group (Table 2). Prevalence rates and differences in the frequency of single care challenges according to the grade of dementia and/or delirium are shown in Table 3. The number of care challenges significantly increased with the severity of cognitive impairment. Cognitively unimpaired patients had a mean of 0.41 care challenges per hospital stay (SD 0.90, range 0–8), patients with mild cognitive impairment a mean of 1.06 (1.55; 0–9), patients with mild dementia and/or delirium a mean of 2.07 (2.10, 0–9), and patients with moderate dementia and/or delirium a mean of 3.82 (2.56, 0–10). Finally, patients with severe dementia and/or delirium had an average of 5.04 care challenges (2.38, 1–11).

**Table 3:** Care challenges among patients with dementia and/or delirium

	Severity of cognitive impairment in hospital									*Significance*	
	Mild dementia and/or delirium CDR^a^ = 1 (*n* = 118)			Moderate dementia and/or delirium CDR^a^ = 2 (*n* = 101)			Severe dementia and/or delirium CDR^a^ = 3 (*n* = 77)			*χ*^*2*^^b^	*p*
	*n*	*%*	*(95% CI)*	*n*	*%*	*(95% CI)*	*n*	*%*	*(95% CI)*		
≥1 care challenge	87	73.7	*65.1–80.8*	95	94.1	*87.6–97.3*	77	100	*95.3–100*	*35.4*	*0.000*
Needing more time than usual	49	41.5	*33.0–50.1*	58	57.4	*47.7–66.6*	61	81.3	*71.1–88.5*	*26.7*	*0.000*
Fending off medical nursing	37	31.4	*23.7–40.2*	67	66.3	*56.7–74.8*	54	71.1	*60.0–80.0*	*36.4*	*0.000*
Fending off/forgetting medication	31	26.3	*19.2–34.9*	56	56.0	*46.2–65.3*	38	50.7	*39.6–61.7*	*22.2*	*0.000*
Fending off wound care	6	5.1	*2.4–10.6*	12	11.9	*6.9–19.6*	18	24.0	*15.8–34.8*	*15.3*	*0.000*
Pulling out infusion needle, catheters etc.	11	9.3	*5.3–15.9*	23	22.8	*15.7–31.9*	25	20	*23.4–44.1*	*16.8*	*0.000*
Limited communication ability	29	24.8	*17.9–33.3*	57	56.4	*46.7–65.7*	69	90.8	*82.2–95.5*	*81.4*	*0.000*
Sleeping disturbance	42	36.2	*28.0–45.3*	40	42.1	*32.7–52.2*	37	50.7	*39.5–61.8*	*3.9*	*0.145*
Fending off basic nursing	16	13.6	*8.5–20.9*	28	28.0	*20.1–37.5*	37	48.7	*37.8–59.7*	*28.6*	*0.000*
Problem with eating and drinking	8	6.8	*3.5–12.8*	24	23.8	*16.5–32.9*	34	45.9	*35.1–57.2*	*40.1*	*0.000*
Leaving food/drinks untouched	5	4.2	*1.8–9.5*	18	17.8	*11.6–26.4*	19	25.3	*16.9–36.2*	*18.2*	*0.000*
Fending off help for eating/drinking	4	3.4	*1.3–8.4*	11	10.9	*6.2–18.5*	25	33.8	*24.1–45.1*	*36.6*	*0.000*
Throwing food	2	1.7	*0.5–6.0*	4	4.0	*1.5–9.7*	5	6.8	*2.9–14.9*	*3.2*^c^	*0.197*^c^
Being verbally agitated	11	9.3	*5.3–15.9*	21	20.8	*15.0–29.7*	26	33.8	*24.2–44.9*	*17.8*	*0.000*
Shouting for help	9	7.6	*4.1–13.9*	20	19.8	*13.2–28.6*	23	29.9	*20.8–40.9*	*14.5*	*0.000*
Insulting others	7	5.9	*2.9–11.7*	11	11.0	*6.3–18.6*	11	14.5	*8.3–24.1*	*4.0*	*0.134*
Complaints from other patients	14	11.9	*7.2–18.9*	20	19.8	*13.2–28.6*	13	16.9	*10.1–26.8*	*2.7*	*0.266*
Ringing bell without recognizable purpose	12	10.2	*5.9–16.9*	19	18.8	*12.4–27.5*	14	18.2	*11.2–28.2*	*3.9*	*0.144*
Problem in physician treatment	8	6.8	*3.5–12.9*	17	17.0	*10.9–25.6*	16	21.6	*13.8–32.3*	*9.3*	*0.010*
Not following physician instructions	7	5.9	*2.9–11.7*	12	12.0	*6.9–20.0*	10	13.9	*7.7–23.7*	*3.8*	*0.148*
Fending off physician treatment	2	1.7	*0.5–6.0*	6	6.0	*2.8–12.5*	12	16.4	*6.5–26.6*	*15.4*	*0.000*
Wandering	11	9.3	*5.3–15.9*	19	18.8	*12.4–27.5*	6	*7.8*	*3.6–16.0*	*6.5*	*0.040*
Being physically aggressive	4	3.4	*1.3–8.4*	13	12.9	*7.7–20.8*	19	24.7	*16.4–35.5*	*19.8*	*0.000*
Leaving ward/hospital unnoticed	5	4.2	*1.8–9.5*	11	10.9	*6.2–18.5*	4	5.3	*2.1–12.8*	*4.2*	*0.126*
Getting injured by fall	3	2.5	*0.9–7.2*	3	3.0	*1.0–8.4*	2	2.6	*0.7–9.0*	*0.2*^c^	*1.00*^c^

^a^*CDR *clinical dementia rating scale

^b^Pearson’s χ^2^-test

^c^Fisher’s exact test

Univariate binary logistic regression analyses revealed statistically significant associations of the presence of ≥1 reported care challenge(s) with dementia and/or delirium, mild cognitive impairment and a lower ADL score (Table 4). With these models the highest proportion (31%) of the variance was explained (Nagelkerke’s R^2^ = 0.31). For the adjusted model all factors were entered simultaneously. Dementia and/or delirium, mild cognitive impairment, lower ADL, getting long-term care benefits, and unplanned admission remained statistically significant predictors. The adjusted model increased the explained variance to 40%. These factors were selected for analysis to examine associations of the different types of care challenges.

**Table 4:** Associations of person-related risk factors with presence of care challenge(s) (≥1): results of univariate and multivariate binary logistic regression analyses

	≥1 reported care challenge					
	Crude OR	95% CI	Nagelkerke’s* R*^*2*^	Fully adjusted OR^a^	95% CI	Nagelkerke’s* R*^*2*^
*Sex*						*0.40*
Female	1.0	Reference	–	1.0	Reference	
Male	0.93	0.78–1.10	*0.00*	0.98	0.82–1.17	
*Age*	**1.06**	**1.04–1.07**	*0.05*	0.99	0.97–1.01	
*Residential situation*						
Community dwelling	1.0	Reference	–	1.0	Reference	
Nursing home	**5.99**	**3.56–10.0**	*0.07*	1.01	0.55–1.88	
*Receiving long-term care (LTC) benefits*						
No/applied for LTC level 0	1.0	Reference	–	1.0	Reference	
Yes	**4.43**	**3.30–5.95**	*0.12*	**1.42**	**1.02–1.96**	
*Cognitive impairment*						
None	1.0	Reference	*0.31*	–	–	
Mild	**2.82**	**2.16–3.67**		**1.98**	**1.50–2.61**	
Dementia and/or delirium	**21.50**	**12.72–36.34**		**7.94**	**4.80–13.13**	
ADL^b^ score (0–85, lower score indicates more need of basic nursing)	**0.95**	**0.95–0.96**	*0.31*	**0.97**	**0.96–0.98**	
Unplanned admission	**1.86**	**1.50–2.30**	*0.03*	**1.44**	**1.06–1.96**	
*Department*						
Internal medicine	1.0	Reference	–	1.0	Reference	
Surgery	0.76	0.56–1.01	*0.00*	0.98	0.79–1.23	
Other	0.95	0.76–1.19	–	1.11	0.83–1.48	

Patients without care challenge constitute reference groups

*OR* odds ratios with *p* < 0.05 in bold

^a^Factors entered simultaneously

^b^*ADL* activities of daily living, modified Barthel index (items bathing and climbing stairs were excluded)

Detailed examination of the impact of dementia and/or delirium on showing ≥1 care challenge(s) revealed a higher risk for patients with mild dementia and/or delirium (odds ratio, OR 4.56, 95% confidence interval, CI 2.68–7.75) and for patients with moderate and severe grades of dementia and/or delirium (OR 25.5; 95% CI 10.1–64.5) compared to the cognitively unimpaired patients. Patients with care challenges showed a high probability for specific care and medical treatment characteristics (Table 5). There was a 70.9 times higher risk for physical restraints and a 4.1 times higher risk for the prescription of psycholeptics. Getting support from relatives in the hospital as well as geriatric/neurological/psychiatric consultations were also more likely for patients with care challenges.

**Table 5:** Associations of medical treatment and care characteristics with presence of care challenge(s) (≥1): results of univariate binary logistic regression analyses

	≥1 reported care challenge		
	Crude OR	95% CI	Nagelkerke’s *R*^*2*^
*Received general anesthesia*			
No	1.0	Reference	–
Yes	0.85	0.67–1.07	0.00
*Physical restraints*^a^			
No	1.0	Reference	–
Yes	**70.92**	**19.66–255.84**	0.13
*Psycholeptics prescription ATC N05*			
No	1.0	Reference	–
Yes	**4.07**	**3.29–5.05**	0.11
*Geriatric/neurological/psychiatric consultation*			
No	1.0	Reference	–
Yes	**2.07**	**1.42–3.04**	0.01
*Analgesic prescription ATC N02*			
No	1.0	Reference	–
Yes	1.07	0.89–1.30	0.00
*Visit of relatives/others*			
None or rare	1.0	Reference	–
Frequent	0.90	0.72–1.11	0.00
*Getting help from relatives in the hospital*			
No	1.0	Reference	–
Yes	**4.95**	**3.21–7.61**	0.08

Patients without care challenge constitute reference groups

*OR* odds ratios with *p* < 0.05 in bold, *CI* confidence interval, *ATC *anatomical therapeutic chemical classification

^a^Unwanted bedside rail and other physical restraints

Analyses of the different types of care challenges revealed that all care challenges were associated with cognitive impairment, in particular with dementia and/or delirium (Table 6). When adjusting for ADL score, unplanned admission and receiving LTC benefits, nearly all care challenges remained significantly associated with dementia and/or delirium. Beside dementia and/or delirium only a lower level of ADL remained an independent risk factor for all care challenges. Similar logistic regression analyses for patients with mild cognitive impairment showed weak but significant associations with some care challenges.

**Table 6:** Association of cognitive impairment, ADL, unplanned hospital admission and receiving long-term care benefits with care challenges: results of univariate and multivariate binary logistic regression analyses

	Crude OR 95% CI			Fully adjusted OR^a^ 95% CI					
	Dementia and/or delirium^b^ CDR 1–3	Mild cognitive impairment^b^ CDR 0.5	Nagelkerke’s* R*^*2*^	Dementia and/or delirium^b^ CDR 1–3	Mild cognitive impairment^b^ CDR 0.5	ADL^c^	Unplanned admission^d^	Getting long-term care benefits^e^	Nagelkerke’s* R*^*2*^
Being physically aggressive	**121.38**	9.20	*0.29*	**28.85**	5.26	**0.97**	**0.52**	1.76	*0.37*
	**17.16–858.76**	0.93–90.85		**2.29–363.33**	0.42–65.95	**0.95–0.99**	**0.28–0.98**	0.76–4.08	
Being verbally agitated	**46.53**	7.02	*0.27*	**13.31**	**4.37**	**0.97**	1.12	1.06	*0.33*
	**18.60–116.40**	2.28–21.61		**4.08–43.43**	**1.23–15.62**	**0.96–0.98**	0.65–1.95	0.56–2.00	
Pulling out infusions needle, catheters etc	**43.57**	3.07	*0.31*	**18.59**	2.24	**0.98**	0.98	0.99	*0.34*
	**16.25–116.79**	0.91–10.40		**6.66–51.86**	0.64–7.80	**0.97–0.99**	0.59–1.65	0.64–1.53	
Wandering	**40.37**	**6.25**	*0.23*	**151.21**	**9.26**	**1.04**	**0.50**	1.24	0.32
	**7.74–184.42**	**1.11–35.05**		**24.86–919.16**	**1.69–50.75**	**1.03–1.06**	**0.26–0.98**	0.57–2.70	
Limited communication ability	**35.46**	**2.10**	*0.42*	**11.52**	1.20	**0.97**	1.13	1.27	*0.49*
	**21.55–58.34**	**1.24–3.56**		**7.11–18.65**	0.69–2.07	**0.96–0.98**	0.71–1.78	0.83–1.93	
Fending off medical nursing	**18.84**	**2.98**	*0.31*	**9.65**	**2.31**	**0.98**	0.99	1.07	*0.35*
	**11.76–30.19**	**2.03–4.39**		**5.87–15.87**	**1.50–3.57**	**0.97–0.99**	0.74–1.34	0.68–1.68	
Fending off wound care	**17.34**	2.19	*0.19*	**8.25**	1.76	**0.98**	0.69	0.81	*0.21*
	**6.64–45.24**	0.76–6.30		**2.69–25.27**	0.59–5.21	**0.97–0.99**	0.39–1.21	2.69–25.3	
Problem with eating and drinking	**16.65**	**4.34**	*0.21*	**3.07**	**2.23**	**0.96**	**0.65**	**1.86**	*0.33*
	**10.31–26.90**	**2.49–7.58**		**1.74–5.42**	**1.89–3.88**	**0.95–0.97**	**0.45–0.93**	**1.14–3.03**	
Needing more time than usual	**15.80**	**2.95**	*0.29*	**5.43**	**1.87**	**0.97**	1.18	1.01	*0.37*
	**10.97–22.74**	**2.19–3.97**		**3.53–8.36**	**1.34–2.61**	**0.96–0.98**	0.86–1.62	0.72–1.60	
Fending off/forgetting medication	**14.58**	**3.02**	*0.25*	**9.01**	**2.52**	**0.99**	0.99	1.03	*0.29*
	**9.54–22.29**	**1.98–4.60**		**5.28–15.38**	**1.58–4.02**	**0.98–0.99**	0.72–1.37	0.67–1.60	
Fending off basic nursing	**12.93**	**2.83**	*0.20*	**4.52**	1.97	**0.97**	0.92	1.12	*0.27*
	**7.73–21.63**	**1.51–5.30**		**2.30–8.88**	0.94–4.13	**0.96–0.98**	0.60–1.42	0.67–1.88	
Leaving food/drinks untouched	**9.55**	**4.08**	*0.13*	**2.25**	**2.41**	**0.97**	0.62	1.55	*0.22*
	**5.71–15.99**	**2.37–7.01**		**1.13–4.48**	**1.42–4.10**	**0.96–0.98**	2.42–0.92	0.90–2.68	
Complaints from other patients	**7.68**	**3.85**	*0.12*	**5.47**	**3.52**	0.99	1.41	0.97	*0.12*
	**4.53–13.04**	**2.13–6.97**		**2.40–12.43**	**1.74–7.12**	0.98–1.00	0.92–2.16	0.57–1.63	
Not following physician instructions	**7.65**	1.65	*0.11*	**11.13**	1.37	1.01	0.81	0.88	0.12
	**3.93–14.88**	0.65–4.18		**3.96–31.26**	0.37–5.15	1.00–1.02	0.43–1.53	0.43–1.81	
Sleeping disturbance	**7.09**	**2.49**	*0.16*	**4.18**	**2.08**	**0.99**	1.36	0.98	*0.18*
	**5.03–10.0**	**1.74–3.56**		**2.91–6.00**	**1.45–2.98**	**0.98–0.99**	0.93–1.97	0.68–1.42	
Getting injured by fall	**6.01**	2.29	*0.06*	4.51	1.45	0.98	**0.15**	1.24	0.10
	**1.79–20.47**	0.42–12.57		0.73–27.87	0.19–11.18	0.96–1.00	**0.02–0.92**	0.37–4.18	
Problem in physician treatment	**5.98**	1.28	*0.13*	**5.57**	1.05	0.99	0.83	1.15	*0.11*
	**3.13–11.42**	0.49–3.31		**2.20–14.12**	0.36–3.10	0.99–1.01	0.47–1.47	0.59–2.23	
Ringing bell without recognizable purpose	**5.65**	**2.74**	*0.09*	2.08	1.89	**0.97**	1.37	0.76	*0.15*
	**3.15–10.12**	**1.45–5.17**		0.86–5.08	0.92–3.87	**0.96–0.98**	0.93–2.00	0.43–1.33	
Fending off physician treatment	**5.00**	1.30	*0.06*	1.59	0.77	**0.98**	0.91	2.33	0.10
	**2.27–9.14**	0.50–3.43		0.51–4.96	0.20–2.96	**0.97–0.99**	0.38–2.18	0.97–5.61	

OR odds ratios with *p* < 0.05 in bold

^a^Factors entered simultaneously

^b^Patients without cognitive impairment (CDR = 0) constitute reference groups

^c^*ADL* activities of daily living score 0–85 points, modified Barthel index (items bathing and climbing stairs were excluded), lower score indicates more need of basic nursing

^d^Patients with planned admission is reference group

^e^Patients without long-term care benefits or care level 0 is reference group

## Discussion

With respect to the study aims it was found that the prevalence rate of one or more reported care challenge(s) in older general hospital patients was 42.0%. This finding confirms the clinical relevance of the concept of care challenges based on everyday experiences of the hospital staff. Furthermore, care challenges in general were closely related to the severity of cognitive impairment. The overall prevalence rate of care challenges increased from 24.6% among cognitively unimpaired patients up to 100% in the group with severe dementia and/or delirium. This emphasizes the enormous impact of cognitive impairment, in particular advanced dementia (with or without delirium) on the care situation. The results are in line with previous studies reporting high rates of adverse outcomes, ranging from more acute healthcare problems (i.e. delirium, falls, pain) and care problems to elevated rates of mortality and institutionalization after discharge [11, 18, 32]. In addition, the findings suggest that mild cognitive impairment already has a detrimental influence on the care situation. Significant relationships with severity of cognitive impairment could be determined for almost all examined single care challenges even after adjustment for ADL score and other significant patient-related factors. Thereby, the present study adds more reliable and detailed information to the as yet limited knowledge of problems in every day hospital routines concerning patients with cognitive impairment [9, 12, 20, 24, 29, 31].

Needing more caring time than usual was reported for 20% of older patients in general and for 57% of the patients with dementia and/or delirium. These findings may justify programs of adjusted staff allocation for care-intensive patients in general hospitals, especially for those with cognitive impairments. In Germany, a legal prerequisite for such targeted improvement of staffing conditions for the most vulnerable groups in acute hospital settings came into force on January 2019 [5].

Besides cognitive impairment, lower ADL level, unplanned hospital admission, and receiving LTC benefits were related to care challenges. These variables might be easily recognized at hospital admission by using simple screening procedures and could serve as risk indicators for upcoming care challenges and health problems. The strong associations of lower ADL level not only with the presence of any care challenge but also with most of specific challenges illustrate the known feeling among nursing staff of being challenged by patients’ noncompliant behaviour with respect to eating/drinking, washing and taking medication [12].

The persistently low but statistically significant association of unplanned admission with the overall presence of care challenges may be due to the severe medical crisis of emergency patients, which is often associated with distress and anxiety up to acute confusion of the patients leading to subsequent care challenges. Furthermore, the study found significant associations between care and medical treatment factors and care challenges. Patients with reported care challenges had a significantly higher risk of being physically restrained and receiving psycholeptics. The prescription of sedative substances is often the only yet insufficient possibility for hospital staff to manage challenging behaviour [12]. Using physical restraints is an undesirable procedure as it may lead to agitation, confusion, functional decline, pressure ulcer, strangulation, death, and adverse psychological effects [16]. The finding that patients with care challenges received more often help and assistance from relatives in the hospital, highlights the value of the triad of patient, staff, and family carers [1].

In general, the results serve to facilitate the effective and broader implementation of approaches which have been proved in model projects to optimize hospital care of vulnerable patient groups [6, 8, 15, 17, 20, 25, 27, 30], and to establish the knowledge of a more person-centered care culture in nonspecialized hospital settings [9, 10]. Indicating frequent, but unsolved problems in the daily care of cognitive impaired patients provides a basis for development of staff training programs. For example, limited ability of patients to communicate their needs and wishes was reported as a frequent care challenge of patients with dementia and/or delirium. In a previous study, the hospital staff themselves underlined the need to enhance the awareness for nonverbal aspects of communication and to apply special communication tools during medical procedures in order to help patients accept the procedures and to avoid or reduce negative consequences [14].

### Strength and limitations

The strength of this study is the large number of older patients from a random sample of general hospitals of two southern states in Germany. In contrast, previous studies examined highly selected groups in single general hospitals, often with special features, such as unplanned hospital stay [13, 32]. A further strength of the study is the use of multiple information sources, including comprehensive interviews with responsible nurses concerning care challenges of each participating patient. In contrast, some previous studies were based on administrative reports of adverse events to the hospital management [32] or expert interviews and staff surveys [12, 24, 31]. Furthermore, interviews were based on the more comprehensive concept of care challenges in everyday care and not only on single adverse events or on the psychiatric concept of behavioral and psychological symptoms of dementia (BPSD) [31].

Generalizability is limited by a priori exclusion of intensive care units, geriatric, neurological, and psychiatric hospitals and units, where reported care challenges may be even more frequent in comparison to other departments. Also, results concerning care challenges are based on the reports of nursing staff and subjected to a number of biases (e.g. social desirability, errors in recognition and judgement).

### Practical recommendations

There is an urgent need to strengthen general hospitals in preventing and managing care challenges, for example by targeted allocation of staff, dementia-friendly and delirium-managing interventions, and specific training programs. Knowledge of risk factors for care challenge helps to identify vulnerable patients and to minimize critical events and secondary harm to patients’ health. Patients with milder cognitive impairment constitute a yet barely recognized patient group, which also confronts hospital staff with care challenges.
